# Peer Review of “Finding Potential Adverse Events in the Unstructured Text of Electronic Health Care Records: Development of the Shakespeare Method”

**DOI:** 10.2196/31547

**Published:** 2021-08-11

*This is a peer-review report submitted for the paper “Finding Potential Adverse Events in the Unstructured Text of Electronic Health Care Records: Development of the Shakespeare Method”*

## Round 1 Review

### General Comments

This study [1] is trying to develop a new method to identify attributed and unattributed potential adverse events (AEs) using the unstructured text of electronic health records (EHRs).

1. After reading the manuscript, I feel the title does not match the study contents. First, the title seems to repeat a fact that is already self-evident.
2. The core of the so-called Shakespeare method is still the latent Dirichlet allocation (LDA) method; I cannot see that any novel methods have been developed.
3. There is no related literature review, as many studies have used LDA methods in EHR data. To really find any AE in unstructured text, natural language processing (NLP) is indispensable.
4. What is the difference between the so-called “Shakespeare method” and LDA topic modeling?
5. What are the three parts in the following statement:
  The Shakespeare method has three parts:
  - Convert each document into a vector of n-gram frequencies.
  - Create two groups of vectors: target and comparison.
  - Trim the n-gram vectors in the target group to those that are significant for the target group.
  - Apply topic analysis to the trimmed target group vectors.
  - Interpret the original documents with topic scores of interest.
6. The description of the method is hard to understand. As stated, “Crucially, events can be described in text but not necessarily attributed to being medical care AEs [14,25,41]; we wanted to develop an unstructured method that would identify them.” What is this unstructured method?

## Round 2 Review

### General Comments

This revision provided more details of the Shakespeare method. However, it seems the authors do not quite understand the alternative method: NLP. This may lead to mistaken conclusions. The questions below need reconsideration.

### Specific Comments

#### Major Comments

1. It is claimed that “Many methods for finding AEs in text rely on predefining possible AEs before searching for prespecified words and phrases or manual labeling (standardization) by investigators.” The dictionary method in the NLP tool could extract most terms, for example, included in the Unified Medical Language System, which can be limited to a “disorder” semantic group as a potential transfusion AE (PTAE) group.
2. The PTAE terms identified through the Shakespeare method actually are a mixture of reasons for transfusion, consequences of the reasons for transfusion, or alternate reasons for PTAEs. The Shakespeare method is not able to identify specific AEs with a causal relationship with transfusion. Then, what is the difference between this method and the NLP dictionary method?
3. It is advisable to include potential use scenarios of the method (eg, will more manual reviews be needed for the results?).

## Abbreviations

AE: adverse event
EHR: electronic health record
LDA: latent Dirichlet allocation
NLP: natural language processing
PTAE: potential transfusion adverse event
